# An Improved Grey Wolf Optimizer Based on Differential Evolution and Elimination Mechanism

**DOI:** 10.1038/s41598-019-43546-3

**Published:** 2019-05-09

**Authors:** Jie-Sheng Wang, Shu-Xia Li

**Affiliations:** 1 School of Electronic and Information Engineering, University of Science & Technology Liaoning, Anshan, 114044 China; 2 National Financial Security and System Equipment Engineering Research Center, University of Science & Technology Liaoning, Anshan, 114044 China

**Keywords:** Learning algorithms, Learning algorithms, Machine learning, Machine learning

## Abstract

The grey wolf optimizer (GWO) is a novel type of swarm intelligence optimization algorithm. An improved grey wolf optimizer (IGWO) with evolution and elimination mechanism was proposed so as to achieve the proper compromise between exploration and exploitation, further accelerate the convergence and increase the optimization accuracy of GWO. The biological evolution and the “survival of the fittest” (SOF) principle of biological updating of nature are added to the basic wolf algorithm. The differential evolution (DE) is adopted as the evolutionary pattern of wolves. The wolf pack is updated according to the SOF principle so as to make the algorithm not fall into the local optimum. That is, after each iteration of the algorithm sort the fitness value that corresponds to each wolf by ascending order, and then eliminate R wolves with worst fitness value, meanwhile randomly generate wolves equal to the number of eliminated wolves. Finally, 12 typical benchmark functions are used to carry out simulation experiments with GWO with differential evolution (DGWO), GWO algorithm with SOF mechanism (SGWO), IGWO, DE algorithm, particle swarm algorithm (PSO), artificial bee colony (ABC) algorithm and cuckoo search (CS) algorithm. Experimental results show that IGWO obtains the better convergence velocity and optimization accuracy.

## Introduction

The swarm intelligence algorithms are proposed to mimic the swarm intelligence behavior of biological in nature, which has become a hot of cross-discipline and research field in recent years. The appearance of swarm intelligent optimization algorithm provides the fast and reliable methods for finding solutions on many complex problems^1,2^. Because the swarm intelligence algorithm have characteristics of self-organization, parallel, distributive, flexibility and robustness, now they have been very widespread used in many cases, such as electric power system, communication network, system identification and parameter estimation, robot control, transportation and other practical engineering problems^3–5^. Therefore, the research on the swarm intelligence optimization algorithms has an important academic value and practical significance.

At present, a variety of swarm intelligence optimization algorithms have been proposed by simulating the biotic population and evolution process in nature, such as particle swarm optimization (PSO) algorithm, shuffled frog leaping algorithm (SFLA), artificial bee colony (ABC) algorithm, ant colony optimization (ACO) algorithm, biogeography-based optimization (BBO) algorithm, and cuckoo search (CS) algorithm. Particle Swarm Optimization (PSO) algorithm put forward by Kennedy and Eberhart to mimic the the foraging behavior of birds and fish flock^6^, but the convergence velocity and searching accuracy of PSO algorithm are unsatisfactory to some extend. Shuffled Frog-leaping Algorithm (SFLA) put forward by Eusuff in 2003 is a novel swarm intelligent cooperative searching strategy based on the natural memetics^7,8^. On the one hand, individuals exchange information in the global searching process, and its search precision is high. On the other hand, SFLA has the disadvantage of slow convergence velocity and easy to falling into the local optimum. Artificial Bee Colony (ABC) Algorithm put forward by Karaboga in 2005 to mimics the finding food source behavior of bees^9^. In order to mimic the social behavior of the ant colony, Dorigo *et al*. Proposed the an novel Ant Colony Optimization (ACO) Algorithm in 2006^10^. But their disadvantages are the slow convergence speed and easy to premature. Biogeography-Based Optimization (BBO) algorithm was put forward by Simon in 2008^11^, whose idea is based on the geographical distribution principle in the biogeography. Cuckoo Search (CS) Algorithm was proposed by Yang and Deb in 2009 based on the cuckoo’s parasitic reproduction mechanism and Levy flights searching strategy^12,13^, whose advantage is that CS algorithm is not easy to fall into the local optimum compared with other intelligent algorithms and has less parameters, and whose disadvantage is that the adding of Levy Flight search mechanism leads to strong leap in the process of search, thus, its local search is not careful.

The common shortcoming of these algorithms is that each swarm intelligence algorithm has problem in different degrees that the convergence velocity is slow, the optimization precision is low, and easy to fall into the local optimum^5^. The key reason cause these shortcoming is that whether an algorithm is able to achieve the proper compromise between exploration and exploitation in its each searching phase or not^14^. Exploration and exploration are contradictory. Exploration reflects the ability of the algorithm to search for new space, while exploration reflects the refining ability of the algorithm. These two criteria are generally used to evaluate stochastic optimization algorithms. Exploration is refers to that a particle leave the original search path in a certain extent and search towards a new direction, which reflects the ability of exploiting unknown regions. Exploitation is refers to that a particle continue to search more carefully on the original trajectory in a certain extent, which can insure the wolf make a detailed search to the region that have been explored. Too small exploration can cause a premature convergence and falling into a local optimum, however, too small exploitation will make the algorithm converge too slowly.

The grey wolf optimizer (GWO) as a novel swarm intelligent optimization algorithm was put forward by Seyedali Mirjalili etc in 2014, which mainly mimics wolf leadership hierarchy and hunting mechanism in nature^15^. Seyedali and Mirjalili etc has proved that the optimization performance of standard GWO is superior to that of PSO, GSA, DE and FEP algorithm. Due to the wolves algorithm with the advantages of simple in principle, fast seeking speed, high search precision, and easy to realize, it is more easily combined with the practical engineering problems. Therefore, GWO has high theoretical research value. But GWO is as a new biological intelligence algorithm, the research about it is just at the initial phase, so research and development of the theory are still not perfect. In order to make the algorithm plays a more superior performance, further exploration and research is needed.

Many swarm intelligence algorithms are mimic the hunting and searching behaviors of some animals. However, GWO simulates internal leadership hierarchy of wolves, thus, in the searching process the position of best solution can be comprehensively assessed by three solutions. But for other swarm intelligence algorithms, the best solution is searched only leaded by a single solution. So GWO can greatly decrease the probability of premature and falling into the local optimum. So as to achieve the proper compromise between exploration and exploitation, an improved GWO with evolution and elimination mechanism is proposed. The biological evolution and the SOF principle of biological updating of nature are added to the basic wolf algorithm. In order to verify the performance of the improved GWO, 12 typical benchmark functions are adopted to carry out simulation experiments, meanwhile, experimental results are compared with PSO algorithm, ABC algorithm and CS algorithm. The experimental results show that the improved grey wolf optimizer (IGWO) obtains the better convergence velocity and optimization accuracy.

The paper is organized as follows. In section 2, the grey wolf optimizer is introduced. A grey wolf optimizer with evolution and SOF mechanism is presented in section 3. In section 4, the simulation experiments are carried out and the simulation results are analyzed in details. Finally, the conclusion illustrates the last part.

## Grey Wolf Optimizer

The grey wolf optimizer is a novel heuristic swarm intelligent optimization algorithm proposed by Seyedali Mirjalili *et al*. in 2014. The wolf as top predators in the food chain, has a strong ability to capture prey. Wolves generally like social life and in the interior of the wolves exists a rigid social hierarchy^15^.

In order to mimic wolves internal leadership hierarchy, the wolves is divided into four types of wolf: *alpha, beta, delta* and *omega*, where the best individual, second best individual and third best individual are recorded as *alpha, beta*, and *delta, and* the rest of the individuals are considered as *omega*. In the GWO, the hunting (optimization) is guided by *alpha, beta*, and *delta*^8^. They guide other wolves (*W*) tend to the best area in searching space. In iterative searching process, the possible position of prey is assessed by three wolves *alpha, beta*, and *delta*. In optimization process, the locations of wolves are updated based on Eqs (1) and (2).

1 $$\overrightarrow{D}=|\overrightarrow{C}\cdot \overrightarrow{{X}_{P}}(t)-\overrightarrow{X}(t)|$$

2 $$\overrightarrow{X}(t+1)=\overrightarrow{{X}_{P}}(t)-\overrightarrow{A}\cdot \overrightarrow{D}$$

where, *t* represents the *t*-th iteration, $\overrightarrow{A}$ and $\overrightarrow{C}$ are coefficient vector, $\overrightarrow{{X}_{P}}$ is the position vector of prey, $\overrightarrow{X}$ represents the wolf position. The vector $\overrightarrow{A}$ and $\overrightarrow{C}$ can be expressed by:

3 $$\overrightarrow{A}=2a\cdot \overrightarrow{{r}_{1}}-\overrightarrow{a}$$

4 $$\overrightarrow{C}=2\cdot \overrightarrow{{r}_{2}}$$

where, the coefficient $\overrightarrow{a}$ linearly decreases from 2 to 0 with the increasing of iteration number, $\overrightarrow{{r}_{1}}$ and $\overrightarrow{{r}_{2}}$ are random vector located in the scope [0, 1].

**Figure 1:**
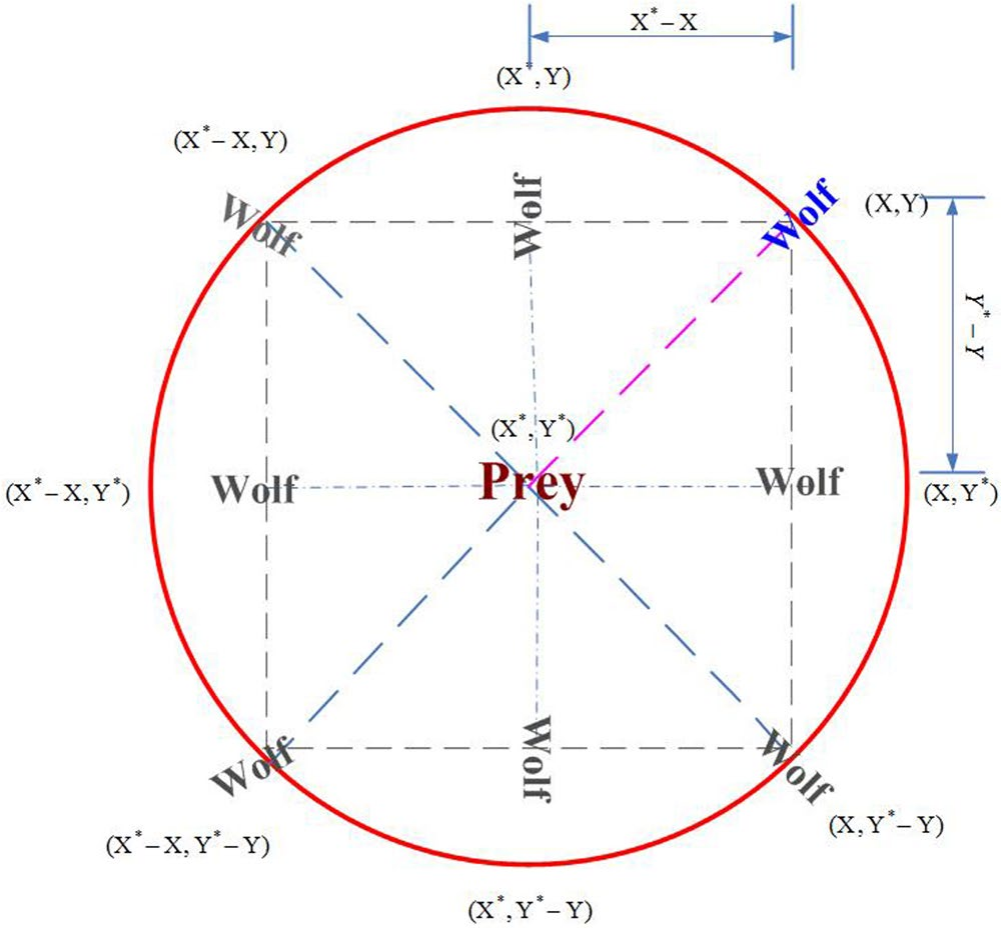
2D position vectors and possible next locations.

**Figure 2:**
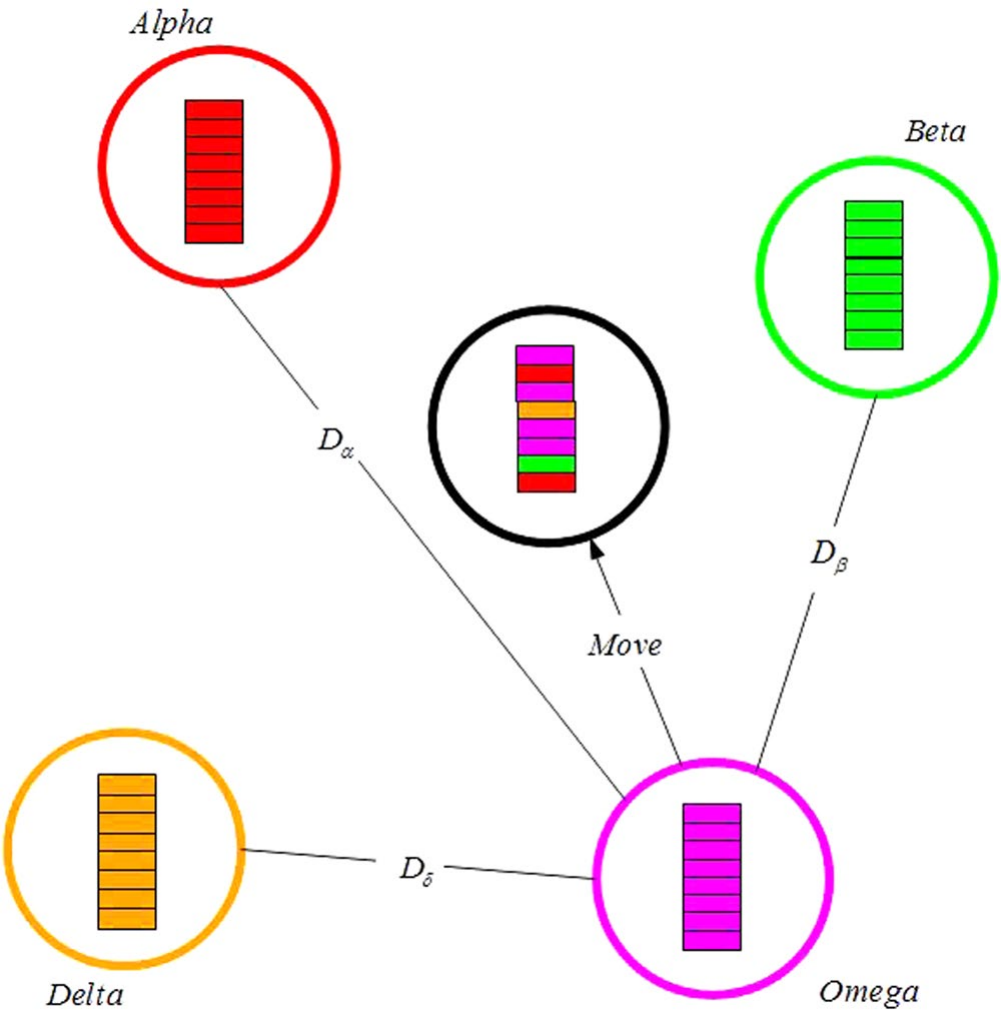
Position updating of IGWO.

Principle of the position updating rules described in Eqs (1) and (2) are shown in Fig. 1. It can be seen from Fig. 1 the wolf at the position (*X*, *Y*) can relocate itself position around the prey according to above updating formulas. Although Fig. 1 only shows 7 positions that the wolf possible move to, by adjusting the random parameters C and A it can make the wolf to relocate itself to any position in the continuous space near prey. In the GWO, it always assumes that position of alpha, beta and delta is likely to be the prey (optimum) position. In the iteration searching process, the best individual, second best individual and third best individual obtained so far are respectively recorded as *alpha, beta*, and *delta. H*owever, other wolves who are regarded as omega relocate their locations according to the locations of *alpha, beta*, and *delta*. The following mathematical formulas are used to re-adjust positions of the wolf *omega*. The conceptual model that wolf update its position is shown in Fig. 2.

5 $${\overrightarrow{D}}_{\alpha }=|\overrightarrow{{C}_{1}}\cdot \overrightarrow{{X}_{\alpha }}-\overrightarrow{X}|$$

6 $${\overrightarrow{D}}_{\beta }=|\overrightarrow{{C}_{2}}\cdot \overrightarrow{{X}_{\beta }}-\overrightarrow{X}|$$

7 $${\overrightarrow{D}}_{\delta }=|\overrightarrow{{C}_{3}}\cdot \overrightarrow{{X}_{\delta }}-\overrightarrow{X}|$$

where, $\overrightarrow{{X}_{\alpha }}$, $\overrightarrow{{X}_{\beta }}$ and $\overrightarrow{{X}_{\delta }}$ are the position vector of *alpha, beta*, and *delta*, respectively. $\overrightarrow{{C}_{1}}$, $\overrightarrow{{C}_{2}}$, $\overrightarrow{{C}_{3}}$ are randomly generated vectors, $\overrightarrow{X}$ represents the position vector of current individual. The Eqs (5), (6) and (7) respectively calculate the distances between the position of current individual and that of individual *alpha, beta*, and *delta*. So the final position vectors of the current individual are calculated by:

8 $$\overrightarrow{{X}_{1}}=\overrightarrow{{X}_{\alpha }}-\overrightarrow{{A}_{1}}\cdot (\overrightarrow{{D}_{\alpha }})$$

9 $$\overrightarrow{{X}_{2}}=\overrightarrow{{X}_{\beta }}-\overrightarrow{{A}_{2}}\cdot (\overrightarrow{{D}_{\beta }})$$

10 $$\overrightarrow{{X}_{3}}=\overrightarrow{{X}_{\delta }}-\overrightarrow{{A}_{3}}\cdot (\overrightarrow{{D}_{\delta }})$$

11 $$\overrightarrow{X}(t+1)=\frac{\overrightarrow{{X}_{1}}+\overrightarrow{{X}_{2}}+\overrightarrow{{X}_{3}}}{3}$$

where, $\overrightarrow{{A}_{1}}$, $\overrightarrow{{A}_{2}}$, $\overrightarrow{{A}_{3}}$ are randomly generated vectors, and *t* represents the number of iterations.

In the plane, three points is able to determine a region. Thus, the scope of position of the prey can be determined by the best three wolves. The GWO that whose target solution is comprehensively assessed by three solutions, can greatly decrease the probability of trapping into the local extreme.

It can be seen from the above formula that Eqs (5–7) respectively define the step size of the omega tend to alpha, beta, and delta. The final positions of the omega wolves are defined by Eqs (8–11).

The exploration ability and exploitation ability have important influence on the searching performance of an algorithm. For the GWO, exploration is refers to a wolf leave the original search path in a certain extent and search towards a new direction, which reflects the wolf’s ability of exploiting unknown regions. Exploitation is refers to that a wolf continue to search more carefully on the original trajectory in a certain extent, which can insure the wolf make a detailed search to the region that have been explored. So how to make the algorithm achieve a proper compromise between exploration and exploitation is a question that worth research.

**Figure 3:**
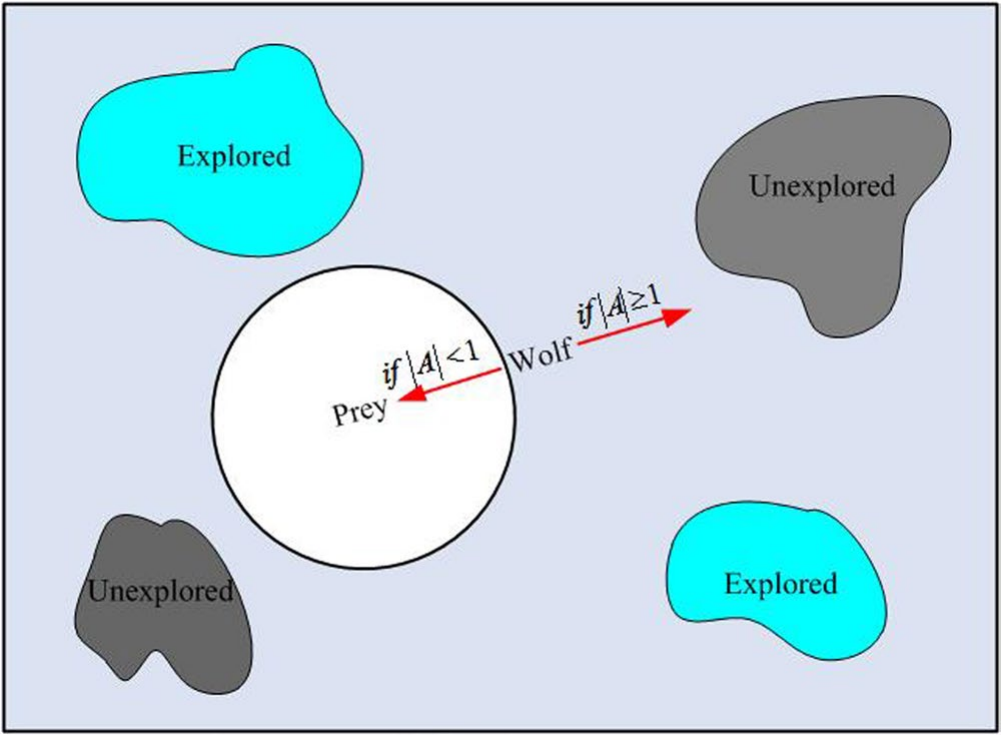
Exploration and exploitation of wolf in GWO.

It can be observed that the two random and adaptive vectors $\overrightarrow{A}$ and $\overrightarrow{C}$ can be used to obtain a proper compromise between exploration ability and exploitation ability of the GWO. As is shown in Fig. 3, when $\overrightarrow{A}$ is greater than 1 and is less than −1, that is $|\overrightarrow{A}| > 1$, the wolf shows exploration ability. When the value of vector $\overrightarrow{C}$ is greater than 1, it can also enhance the exploration ability of the wolf. In contrast, when $|\overrightarrow{A}| < 1$ and *C* < 1 the wolf’s exploitation capacity is enhanced. For increasing the exploitation ability of the wolf gradually, the vector $\overrightarrow{A}$ decreases linearly with the iterations number increasing. However, in the course of optimization the value of $\overrightarrow{C}$ is generated randomly, which can make exploration and exploitation of the wolf reach a equilibrium at any stage. Especially in the final stages of the iteration, it is able to avoid the algorithm from trapping into a local extreme. The pseudo codes of the GWO are described as follows.

Initialize the population *X*_*i*_(*i* = 1, 2, … *n*) of GWO

Initialize GWO parameters (*a*, A, C)

Calculate the individual fitness value in the population

Record the best individual, second best individual and third best individual as $\overrightarrow{{X}_{\alpha }}$, $\overrightarrow{{X}_{\beta }}$ and $\overrightarrow{{X}_{\delta }}$

While (*t*< maximum iteration number)

For each individual

Update the position of current individual by Eqs (5–11)

End for

Update *a*, A, C

Calculate the fitness value of all individual in the population

Update $$\overrightarrow{{X}_{\alpha }}$$, $$\overrightarrow{{X}_{\beta }}$$, $$\overrightarrow{{X}_{\delta }}$$

*t* = *t* + 1

End While

Return $$\overrightarrow{{X}_{\alpha }}$$

The GWO has strong exploration ability, which can avoid the algorithm falling into the local optimum. For the GWO, the proper compromise between exploration ability and exploitation ability is very simple to be achieved, so it can effectively solve many complicated problems.

## Improved Grey Wolf Optimizer (IGWO)

For increasing the search performance of the GWO, an improved grey wolf optimizer (IGWO) is proposed. In the IGWO, the biological evolution and the SOF principle of biological updating of nature are added to the standard GWO. Due to the differential evolution algorithm having the advantages of simple principle, less algorithm parameters and easy implementation, differential evolution (DE) strategy is chose as the evolutionary pattern of wolves in this paper. The wolf pack is updated according to the SOF principle so as to make the algorithm not fall into the local optimum. That is, after each iteration of the algorithm sort the fitness value that corresponds to each wolf by ascending order, and then eliminate R wolves with larger fitness value, meanwhile randomly generate wolves that equal to the number of eliminated wolves.

### Grey Wolf Optimizer with evolution operation

In nature, organisms evolve from the low level to advanced level continually under the action of heredity, selection and mutation^16,17^. Similarly, there are also a series of changes like heredity, selection and mutation in the searching process of the wolves. The evolution law of SOF make wolves gradually strong. In the same way, for increasing the searching performance of the algorithm, the evolution operation is added to basic GWO. Based on the biological evolution of nature, many evolution methods have been developed, such as differential evolution(DE), quantum evolution and cooperative evolution, etc.^18^. For all the evolution methods, the differential evolution strategy has simple principle, less parameters and easy implementation and it has been extensively researched and applied^19,20^. Therefore, the DE strategy is chose as the evolution method of GWO. The basic principle of DE operator is to adopt the difference among individuals to recombine the population and obtain intermediate individuals, and then get the next generation population through a competition between parent individual and offspring individual^21,22^. The basic operations of DE include three operations: mutation, crossover and selection. After the operation of evolution is added to GWO, the wolf’s position updating is shown as Fig. 2.

#### Mutation operation

The most prominent feature of differential evolution is mutation operation. When an individual is selected, two differences with weight are added to the individual to accomplish its variation^23^. The basic variation ingredient of DE is the difference vector of the parents, and each vector contains two different individuals $({X}_{r1}^{t},{X}_{r2}^{t})$ of parent (the *t-th* generation). The difference vector is defined as follows.

12 $${\rm{D}}{d}_{r12}={X}_{r1}^{t}-{X}_{r2}^{t}$$

where, *r*_1_ and *r*_2_ express index number of two different individuals of the population. Thus the mutation operation can be described as:

13 $${V}_{i}^{t+1}={X}_{r3}^{t}+F\ast ({X}_{r1}^{t}-{X}_{r2}^{t})$$

where, *r*_1_, *r*_2_ and *r*_3_ are different integers in the scope (1, 2, … *n*) from the current target vector index *i*. F is the scaling factor to control the scaling of differential vector.

In order to produce an ideal variation factor, to ensure that wolves can evolve toward the direction that good for development of the wolves. So in this paper, chooses outstanding individuals of wolves as parents. After a large number of simulation experiments, beta and delta are chose as two parents, and then combined with the alpha wolf to form a variation factor. Therefore, the variation factor is designed as Eq. (14).

14 $${V}_{i}^{t+1}={X}_{\alpha }^{t}+F\ast ({X}_{\beta }^{t}-{X}_{\delta }^{t})$$

In order to make the algorithm has a high exploration ability in the early stage to avoid falling into local optimum, and has a high exploitation ability in the latter stage to increase the convergence speed, a dynamic scaling factor is employed. So scaling factor *F* change from large to small according to the iteration number in Eq. (15).

15 $${\rm{F}}={f}_{{\rm{\min }}}+({f}_{{\rm{\max }}}-{f}_{{\rm{\min }}})\times \frac{Max\_iter-(iter-1)}{Max\_iter}$$

where, *f*_min_ and *f*_max_ are the minimum and maximum of the scaling factor, *Max*_*iter* is maximum iteration number. *iter* is the *iter*-th iteration number.

#### Crossover operation

For the target vector individual ${X}_{i}^{t}$ of the wolves, make it have a crossover operation with the variation vector ${V}_{i}^{t+1}$, and produce a test individual ${U}_{i}^{t+1}$. In order to guarantee the individual ${X}_{i}^{t}$ taking place a evolution, a random choice method is adopt to insure at least one bit of ${U}_{i}^{t+1}$ is contributed by ${U}_{i}^{t+1}$. For other bits of ${U}_{i}^{t+1}$, the crossover probability factor CR is used to decide which bit of ${U}_{i}^{t+1}$ is contributed by ${V}_{i}^{t+1}$, and which bit is contributed by ${X}_{i}^{t}$. Crossover operation is express as follows.

16 $${U}_{ij}^{t+1}=\{\begin{array}{cc}{V}_{ij}^{t+1} & rand(j)\le CR\,or\,j=randn(i)\\ {X}_{ij} & rand(j)\ge CR\,and\,j\ne randn(i)\end{array}\,j=1,2,\cdots ,D$$

where, *rand*(*j*) ∈ [0, 1] obeys the random-uniform distribution, *j* is the *j*-th variable (gene), *CR* is crossover probability, and *rand*(*i*) ∈ [1, 2, … D].

It can be known from the Eq. (16), if the *CR* is larger, ${V}_{i}^{t+1}$ is able to make more contribution to ${U}_{i}^{t+1}$. When *CR* = 1, ${U}_{i}^{t+1}={V}_{i}^{t+1}$. If the *CR* is smaller, ${X}_{i}^{t}$ is able to make more contribution to ${U}_{i}^{t+1}$.

#### Selection operation

The “greedy choice” strategy is applied to the selection operation. After mutation operation and crossover operation generate the experiment individual ${U}_{i}^{t+1}$ and the compete it with ${X}_{i}^{t}$. It can be expressed as Eq. (17).

17 $${X}_{i}^{t+1}=\{\begin{array}{c}{U}_{i}^{t+1},\\ {X}_{i}^{t},\end{array}\,\begin{array}{c}f({U}_{i}^{t+1}) < f({X}_{i}^{t})\\ f({U}_{i}^{t+1})\ge f({X}_{i}^{t})\end{array}\,\,i=1,2,\cdots \,n$$

where, *f* is the fitness function, ${X}_{i}^{t+1}$ is the individual of *t*-th generation. from ${U}_{i}^{t+1}$ and ${X}_{i}^{t}$ choose the individual with best fitness as an individual of *(t* + *1)-* generation, and replace the individual of the *t-th* generation.

In the early stages of the algorithm, difference of the population is large, so mutation operation makes the algorithm has strong exploration ability. In the later stages of the algorithm iteration, namely when the algorithm tends to converge, difference between individuals of the population is small, which makes the algorithm has a strong exploitation ability.

### SOF wolves updating mechanism

SOF is a role of nature that formed in the process of the biological evolution^23–25^. In nature, some vulnerable wolves will be eliminated because of uneven distribution of the prey, hunger, disease and other reasons. Meanwhile, new wolves will join to this wolf organization to enhance fighting capacity of the organization, which can insure the wolf organization survival well in the complicated world. The wolf pack is updated according to the SOF principle so as to make the algorithm not fall into the local optimum^26–29^.

In the new algorithm, assume the number of wolves in the pack is fixed, and the strength of wolves is measured by fitness value. The higher the fitness, the better the solution. Therefore, after each iteration of the algorithm sort the fitness value that corresponds to each wolf in ascending order, and then eliminate *R* wolves with larger fitness value, meanwhile randomly generate new wolves that equal to the number of eliminated wolves. When R is large, the number of wolves that new generating is big, which will help to increase the diversity of wolves. But if the value of *R* is too large, the algorithm tends to be searching randomly, which will results in the convergence speed becoming slow. If the value of *R* is too small, it is not conducive to maintain the diversity of population, which results in the ability of exploring new solution space weakened. Therefore, in this paper, *R* is a random integer between *n*/(2 × *ε*) and *n*/*ε*.

18 $$R=[n/\varepsilon ,n/0.75\times \varepsilon ]$$

where, *n* is the total number of wolves, *ε* is scale factor of wolves updating.

**Figure 4:**
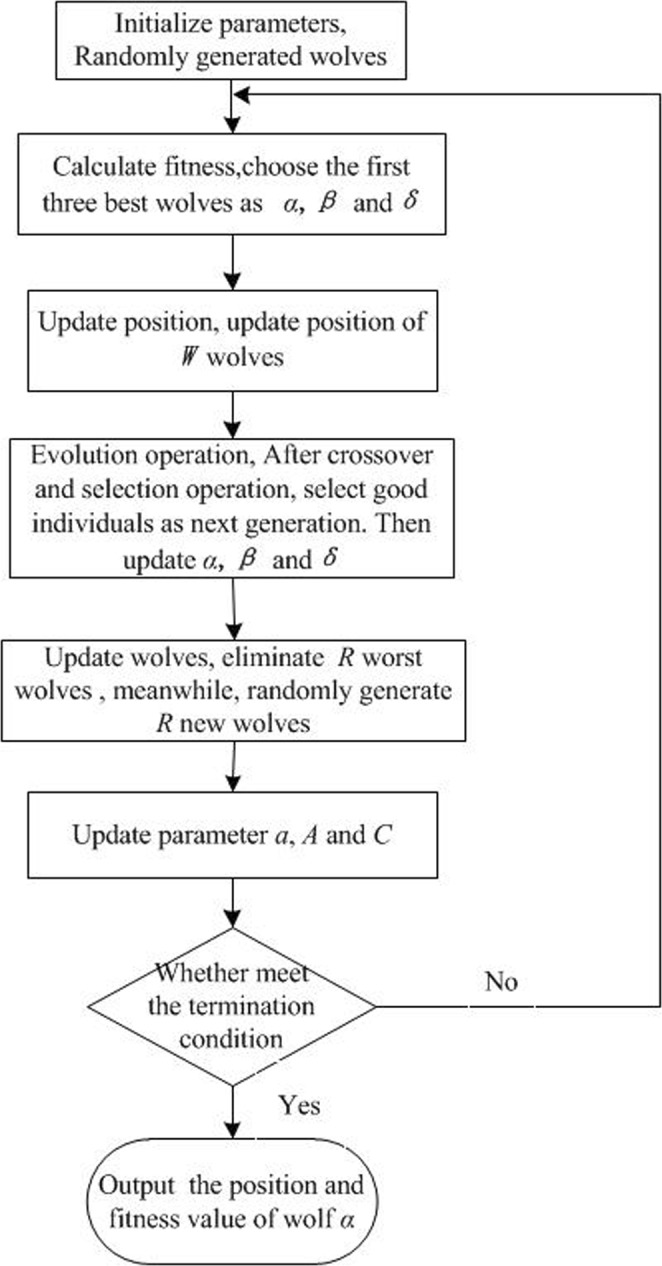
Flow chart of IGWO algorithm.

The flow chart of the improved grey wolf optimizer (IGWO) is illustrated in Fig. 4. The main procedure steps are described as follows.

1. Initialize the grey wolf population. Randomly generated position of wolves *X*_*i*_(*i* = 1, 2, … *n*) Initialize parameters *a*, A and C.
2. Calculate fitness of each wolf, choose the first three best wolves and save them as alpha beta and Delta in turn.
3. Update position. According to Eqs (5–11) update position of the other wolves, that is, update position of omega wolf.
4. Evolution operation. Use alpha, beta, and delta to form variation factor according to the Eq. (14). After crossover and selection operation, select the individual with good fitness as the wolf of next generation. Choose the first three best wolves and save them as alpha beta and Delta in turn.
5. Update wolves. Sort fitness values that correspond by wolves from small to large, eliminate R wolves with larger fitness value. Meanwhile, randomly generate new R wolves.
6. Update parameter *a*, A and C.
7. Judge whether the termination condition is satisfied, if satisfied, output the position and fitness value of alpha as the optimal solution. If not satisfied, return to step (2).

## Simulation Experiments and Results Analysis

Before carrying out the simulation experiments to compare the performances of adopted optimization algorithm, twelve benchmark functions are selected^2^, which are listed in Table 1. The experiment consists of two parts. For validating the performance of two improvements to the GWO, one part is that separately do experiments for the GWO with differential evolution (DGWO), GWO with SOF mechanism (SGWO) and IGWO. And meanwhile, compare results with DE algorithm. The second part is that do experiments to compare IGWO with other swarm intelligence algorithm, including PSO algorithm, ABC algorithm and CS algorithm. The parameter settings of GWO, PSO algorithm, ABC algorithm, CS algorithm and, DE algorithm are defined according with literature and can be found respectively in^8,9,12,13,23^, which are listed in Table 2.

**Table 1:** Test functions.

Function name	Function	Rang	F_min_
Sphere	${f}_{1}(x)=\sum _{i=1}^{d}{x}_{i}^{2}$	[−100, 100]	0
Sumsquares	${f}_{2}(x)=\sum _{i=1}^{d}i{x}_{i}^{2}$	[−10, 10]	0
Schwefel	${f}_{3}(x)={\sum _{i=1}^{d}({\sum }_{j-1}^{i}{x}_{j})}^{2}$	[−100, 100]	0
Schwefel 2.21	${f}_{4}(x)={{\rm{\max }}}_{i}\{|{x}_{i}|,1\le i\le n\}$	[−100, 100]	0
Rosenbrock	${f}_{5}(x)=\sum _{i=1}^{d-1}(100({x}_{i+1}-{x}_{i}^{2})+{({x}_{i}-1)}^{2})$	[−30, 30]	0
step	${f}_{6}(x)={\sum }_{i=1}^{d}{([{x}_{i}+0.5])}^{2}$	[−100, 100]	0
Quartic	${f}_{7}(x)={\sum }_{i=1}^{d}i{x}_{i}^{4}+random[0,1)$	[−1.28, 1.28]	0
Schwefel	${f}_{8}(x)={\sum }_{i=1}^{n}-\,{x}_{i}\,\sin (\sqrt{{x}_{i}})$	[−500, 500]	−418.9 × 5
Rastrigrin	${f}_{9}(x)=\sum _{i=1}^{d}({{x}_{i}}^{2}-10\,\cos (2\pi {x}_{i})+10)$	[−5.12, 5.12]	0
Ackley	${f}_{10}(x)=-\,20\,\exp (-0.2\sqrt{\frac{1}{n}{\sum }_{i=1}^{n}{x}_{i}^{2}})-\exp (\frac{1}{n}{\sum }_{i=1}^{n}\cos (2\pi {x}_{i}))+20+e$	[−32, 32]	0
Griewank	${f}_{11}(x)=\frac{1}{4000}(\sum _{i=1}^{n}({x}^{2}i))-(\prod _{i=1}^{n}\cos (\frac{xi}{\sqrt{i}}))+1$	[−600, 600]	0
Penalty#1	$$\begin{array}{c}{f}_{12}(x)=\frac{\pi }{n}\{10\,\sin (\pi {y}_{1})+{\sum }_{i=1}^{n-1}{({y}_{i}-1)}^{2}[1+10{\sin }^{2}(\pi {y}_{i+1}]+{({y}_{n}+1)}^{2}\}\\ \,+{\sum }_{i=1}^{n}u({x}_{i},10,100,4)\end{array}$$ $$\begin{array}{c}{y}_{i}=1+\frac{{x}_{i}+1}{4}\\ u({x}_{i},a,k,m)=\{\begin{array}{c}k{({x}_{i}-a)}^{m}\\ 0\\ k{(-{x}_{i}-a)}^{m}\end{array}\begin{array}{c}{x}_{i} > a\\ -a < {x}_{i} < a\\ {x}_{i} < -a\end{array}\end{array}$$	[−50, 50]	0

**Table 2:** Parameters Settings of each algorithms.

Algorithm	Main parameters Settings
PSO	Particle number *n* = 30; Learning factor *c1* = 2, *c2* = 2; Inertia weight *w* = 0.9
ABC	Bees number *n* = 30; Follow-bees number *n/2* = 10; Lead-bees number *n*/*2* = 10
CS	Bird nest number *n* = 30; Detection probability*Pa* = 0.25; Step length control *α* = 0.01
DE	population size *n* = 30; CR = 0.7; F = 1
GWO	Wolves number *N* = 30
DGWO	Wolves number *N* = 30; f_min_ = 0.25, f_max_ = 1.5; CR = 0.7
SGWO	Wolves number *N* = 30; *ε* = 5
IGWO	Wolves number *N* = 30; f_min_ = 0.25, f_max_ = 1.5; CR = 0.7; *ε* = 5

### Experiment and analysis for two improvement of IGWO

**Figure 5:**
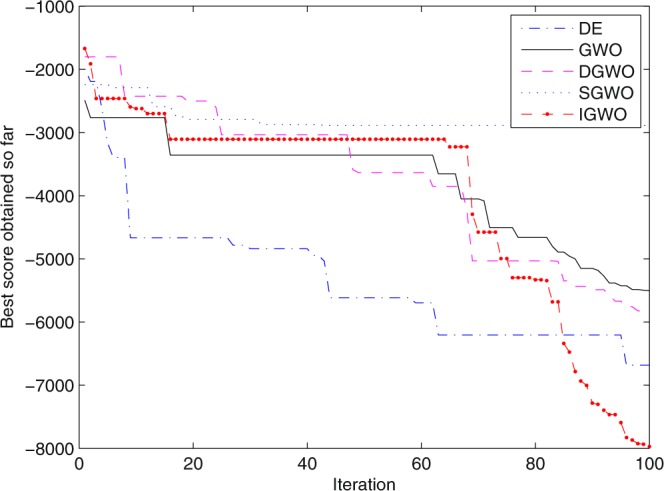
Convergence curves Function *F8* (D = 30).

**Figure 6:**
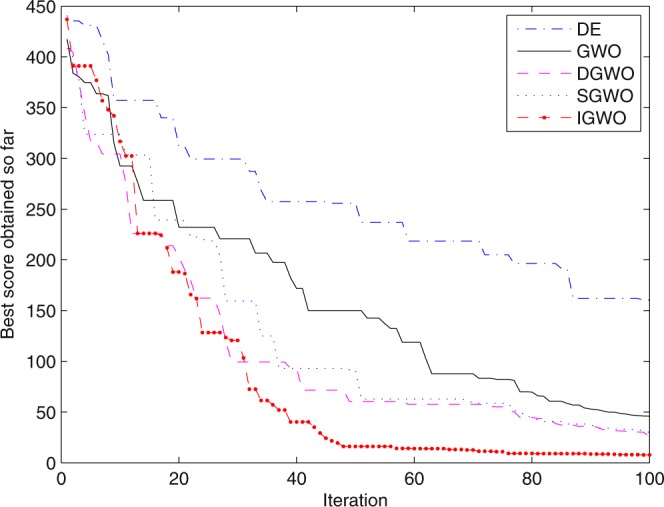
Convergence curves Function *F9* (D = 30).

**Figure 7:**
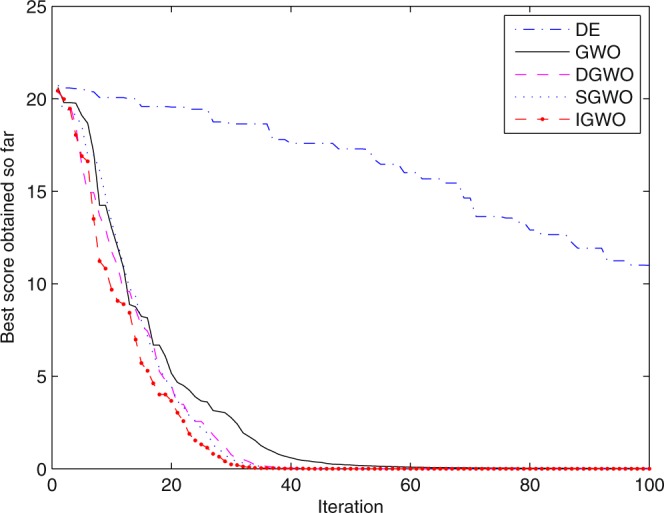
Convergence curves Function *F10* (D = 30).

**Figure 8:**
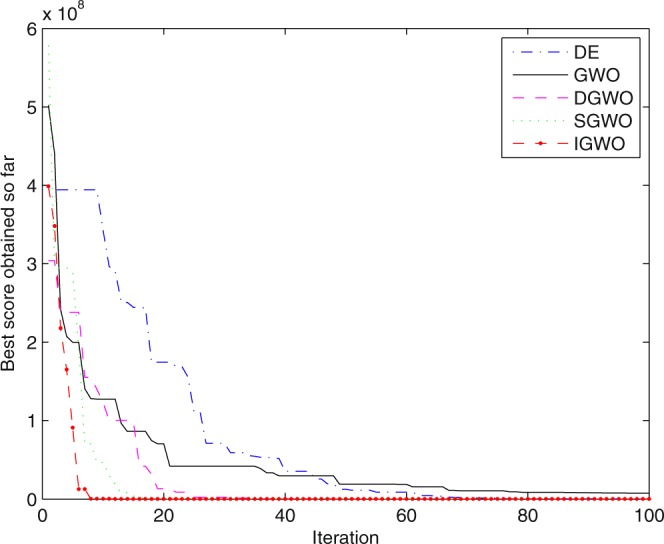
Convergence curves Function *F12*_._ (D = 30).

**Figure 9:**
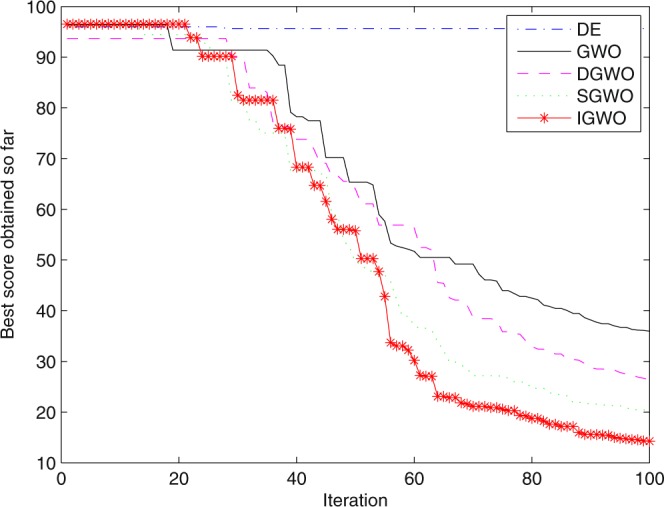
Convergence curves Function *F4*_._ (D = 100).

**Figure 10:**
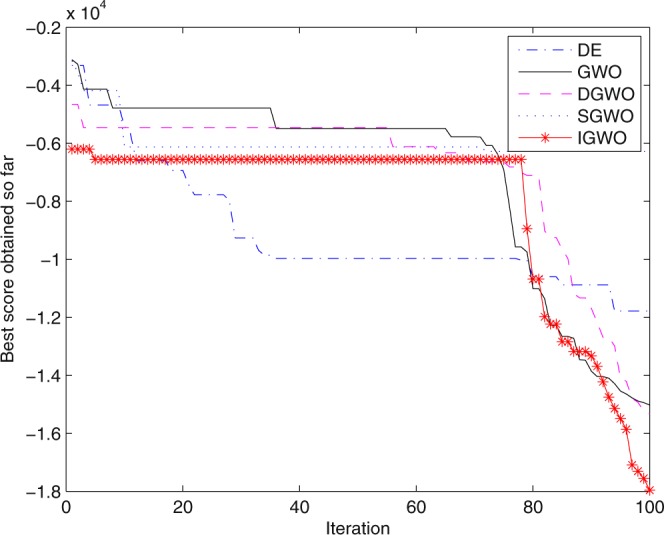
Convergence curves Function *F8* (D = 100).

**Figure 11:**
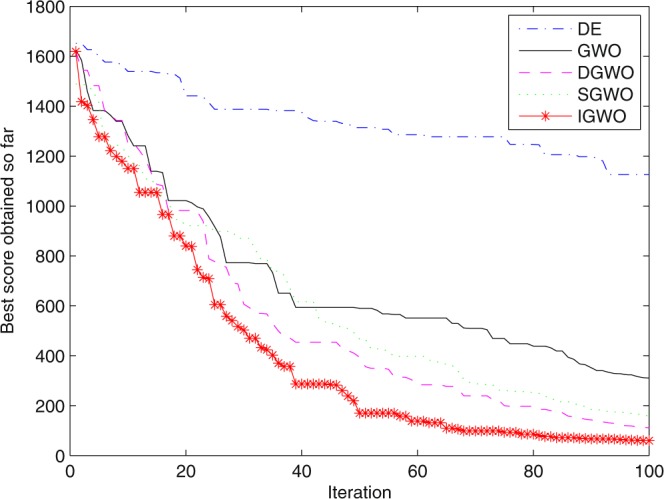
Convergence curves Function *F9* (D = 100).

For validating the performance of two improvements to the GWO, first of all, simulation experiments are separately carried out for the grey wolf optimizer with differential evolution (DGWO), grey wolf optimizer with SOF mechanism (SGWO) and IGWO. And meanwhile, The simulation results compared with GWO and DE algorithm are shown in Figs 5–11. It can be seen from the simulation convergence curves for the adopted testing functions, compared with GWO, the convergence velocity and optimization precision of DGWO, SGWO and IGWO all have been improved, but IGWO is the best.

Then for further validating the searching accuracy of DGWO, SGWO and IGWO, every optimization algorithm is run independently thirty times and the best, worst and average values are recorded for the adopted twelve testing functions under the 30-dimension and 100-dimension. The maximum iterations number is *Max Max_iter* = 500. The statistical results of D = 30 and D = 100 are shown in Tables 3 and 4 respectively.

**Table 3:** Numerical statistics results of D = 30.

Function		IGWO	SGWO	DGWO	GWO	DE
**F1**	Best	**3.9273e** **−** **069**	4.2813e−064	1.3776e−066	2.0291e−029	2.8252e−004
	Ave	1.1783e **−** 064	8.6129e **−** 061	4.3208e **−** 062	1.0402e **−** 027	5.4593e **−** 004
	Worst	1.2505e **−** 062	1.0843e **−** 059	3.2370e **−** 061	6.8810e **−** 027	0.0010
	Std	5.5606e **−** 063	8.4600e **−** 060	1.4297e **−** 061	1.4561e **−** 027	3.7365e **−** 004
**F2**	Best	**7.6878e** **−** **068**	1.3615e **−** 048	3.9913e **−** 056	2.0613e **−** 030	0.0015
	Ave	3.2484e **−** 064	4.5866e **−** 043	5.7834e **−** 055	6.5600e **−** 029	0.0022
	Worst	8.4385e **−** 063	7.2218e **−** 038	6.8134e **−** 041	4.8634e **−** 028	0.0031
	Std	4.1566e **−** 066	2.4788e **−** 061	5.4083e **−** 059	1.5256e **−** 028	0.0017
**F3**	Best	**1.7846e** **−** **015**	6.9725e **−** 012	1.9482e **−** 013	2.6272e **−** 008	2.3690e + 004
	Ave	3.6220e **−** 0010	4.8496e **−** 008	3.1035e **−** 08	1.4089e **−** 005	2.3690e + 004
	Worst	5.5878e **−** 008	1.3048e **−** 006	8.3608e **−** 07	1.7721e **−** 004	4.0729e + 004
	Std	4.3091e **−** 008	1.0902e **−** 007	1.0736e **−** 006	5.1567e **−** 005	3.8734e + 003
**F4**	Best	**4.0775e** **−** **014**	1.6260e **−** 013	1.5917e **−** 013	8.7151e **−** 008	8.9197
	Ave	4.2932e **−** 013	2.7047e **−** 011	7.9211e **−** 012	1.0129e **−** 006	12.5708
	Worst	7.1302e **−** 011	1.7101e **−** 010	1.0844e **−** 011	7.7390e **−** 006	16.8491
	Std	3.7329e **−** 012	3.7329e **−** 012	7.9664e **−** 011	3.5107e **−** 007	2.5643
**F5**	Best	**23.1787**	26.0680	25.3311	25.7961	46.7705
	Ave	25.3873	27.0246	27.7795	28.0325	147.9340
	Worst	28.5692	28.7589	28.7480	28.7800	229.5079
	Std	0.6997	0.8119	0.8723	0.9258	156.3534
**F6**	Best	**7.5296e** **−** **005**	0.3979	0.2487	0.0063	0.5679
	Ave	0.6583	0.9671	0.7544	0.8993	0.7668
	Worst	2.1169	1.7347	1.5054	1.5192	1.1668
	Std	0.2917	0.3500	0.3096	0.4055	0.3643
**F7**	Best	**1.2695e** **−** **004**	3.7651e **−** 004	8.5420e **−** 004	4.5944e **−** 004	0.0343
	Ave	7.8361e **−** 004	0.0012	9.1816e **−** 004	0.0022	0.0544
	Worst	0.0017	0.0050	0.0011	0.0064	0.0761
	Std	3.7904e **−** 004	4.0563e **−** 004	4.0635e **−** 004	8.4619e **−** 004	0.0356
**F8**	Best	**−3.9429e + 004**	−8.1054e + 003	−7.3195e + 003	−6.1310e + 003	−1.1275e + 004
	Ave	−9.0105e + 003	−4.7468e + 003	−5.8337e + 003	−3.6813e + 003	−7.0046e + 003
	Worst	−3.3482e + 003	−3.5050e + 003	−3.1866e + 003	−2.9262e + 003	−3.8532e + 003
	Std	−1.7912e + 003	−1.2625e + 003	−1.4600e + 003	−958.0854	−5.4353e + 003
**F9**	Best	**0**	**0**	**0**	1.1369e **−** 013	59.0260
	Ave	1.0783	1.2274	1.1754	3.2143	85.4876
	Worst	12.1696	9.2887	12.5246	15.8356	98.3991
	Std	1.5038	2.3349	2.6704	4.8809	56.1783
**F10**	Best	**1.5099e** **−** **017**	1.1546e **−** 014	1.5099e **−** 014	7.5495e **−** 013	0.0035
	Ave	1.2204e **−** 016	2.0073e **−** 014	1.7468e **−** 014	1.0048e **−** 012	0.0055
	Worst	2.2204e **−** 014	2.5757e **−** 014	2.2204e **−** 014	1.4655e **−** 013	0.0082
	Std	4.3110e **−** 015	4.7283e **−** 014	5.4372e **−** 014	1.4373e **−** 014	0.0025
**F11**	Best	**0**	**0**	**0**	**0**	5.3482e **−** 004
	Ave	0.0016	0.0105	0.0060	0.0048	0.0057
	Worst	0.0147	0.0184	0.0441	0.0286	0.0271
	Std	0.0074	0.0085	0.0101	0.0114	0.0432
**F12**	Best	**0.0065**	0.0192	0.0189	0.0188	0.0942
	Ave	0.0481	0.0650	0.0535	0.0594	0.1663
	Worst	0.1091	0.0769	0.1426	0.0819	0.2087
	Std	0.0151	0.0161	0.0318	0.0419	0.0426

**Table 4:** Numerical statistics results of D = 100.

Function		IGWO	SGWO	DGWO	GWO	DE
**F1**	Best	**1.5561e** **−** **035**	3.0615e **−** 034	1.8785e **−** 034	3.4355e **−** 013	1.5008e + 003
	Ave	9.5901e **−** 034	5.6751e **−** 032	1.1231e **−** 032	1.4097e **−** 012	1.8397e + 003
	Worst	1.8708e **−** 032	8.7514e **−** 031	6.8749e **−** 032	3.6831e **−** 012	2.3330e + 003
	Std	7.1710e **−** 034	8.0479e **−** 034	9.9774e **−** 034	2.8268e **−** 012	5.5463e + 003
**F2**	Best	**2.4553e** **−** **036**	6.3490e **−** 034	8.1124e **−** 035	1.6794e **−** 013	98.3138
	Ave	7.1368e **−** 034	2.1103e **−** 031	9.6300e **−** 032	1.0558e **−** 012	4.3298e + 003
	Worst	5.9594e **−** 033	2.9801e **−** 030	1.2238e **−** 032	4.7932e **−** 012	1.2236e + 004
	Std	1.6942e **−** 034	3.2532e **−** 034	3.9658e **−** 034	3.1877e **−** 012	1.4523e + 004
**F3**	Best	**23.6476**	43.0528	25.3814	65.1383	3.5233e + 005
	Ave	679.3675	1.6097e + 003	690.0015	818.9336	4.1657e + 005
	Worst	2.5006e + 003	1.1074e + 004	719.8134	5.5568e + 003	4.7246e + 005
	Std	1.0607e **−** 009	1.4133e **−** 008	1.2798e **−** 007	3.0654e **−** 005	3.4534e **−** 004
**F4**	Best	**0.0018**	0.0044	0.0035	0.0607	86.3861
	Ave	0.0382	0.4416	0.1585	0.9562	90.0565
	Worst	0.6247	2.5503	1.6033	3.1841	92.5876
	Std	0.4072	0.4114	0.6602	0.6513	0.5346
**F5**	Best	**95.8954**	96.0948	97.0946	96.8501	1.4284e + 006
	Ave	96.8157	97.8475	97.5528	98.0217	2.1571e + 006
	Worst	98.5244	98.5851	98.4426	98.5207	3.7347e + 006
	Std	0.6162	0.6385	0.6579	0.6708	1.4353e + 005
**F6**	Best	**7.4830**	10.3930	8.7155	9.7480	1.2876e + 003
	Ave	9.3215	12.0996	11.2379	12.1379	1.7987e + 003
	Worst	12.6824	13.8231	11.7620	12.7860	2.3616e + 003
	Std	0.7722	0.7882	0.9711	0.9744	1.2354e + 003
**F7**	Best	**3.5264e** **−** **004**	0.0013	6.4848e **−** 004	0.0026	2.2367
	Ave	0.0024	0.0089	0.0077	0.0106	3.4390
	Worst	0.0043	0.0581	0.0240	0.0430	7.1933
	Std	7.0880e **−** 004	9.1433e **−** 004	0.0010	0.0021	2.3451
**F8**	Best	**−8.2275e + 004**	−8.6793e + 003	−1.9539e + 004	−1.9447e + 004	−1.8209e + 004
	Ave	−6.0161e + 004	−7.1528e + 003	−1.6209e + 004	−1.5641e + 004	−1.6567e + 004
	Worst	−5.0981e + 003	−6.3427e + 003	−6.4163e + 003	−5.5875e + 003	−1.5388e + 004
	Std	672.4919	2.3324e + 003	3.3000e + 003	4.6259e + 003	2.3453e + 003
**F9**	Best	**0**	**0**	**0**	4.8431e **−** 011	754.1063
	Ave	1.6643	5.7767	4.6137	9.4635	805.4216
	Worst	12.7963	14.7901	9.4707	30.0252	860.7482
	Std	2.6605	3.2557	6.3543	6.4071	764.3451
**F10**	Best	**6.4837e** **−** **014**	6.8390e **−** 014	6.7837e **−** 014	5.9873e **−** 008	6.7720
	Ave	7.7153e **−** 014	8.9943e **−** 014	8.5771e **−** 014	1.1140e **−** 007	7.4253
	Worst	9.3259e **−** 014	1.1102e **−** 013	8.9153e **−** 014	2.2161e **−** 007	9.0925
	Std	6.0990e **−** 015	1.0364e **−** 014	9.9761e **−** 015	6.2626e **−** 008	8.3465
**F11**	Best	**0**	**0**	**0**	1.4655e **−** 013	6.2536e **−** 004
	Ave	0.0028	0.0057	0.0042	0.0065	0.0091
	Worst	0.0254	0.0702	0.0748	0.0270	0.0420
	Std	0.0076	0.0082	0.0090	0.0110	0.0432
**F12**	Best	**0.1056**	0.1706	0.2034	0.2154	6.4979e + 005
	Ave	0.1551	0.3535	0.3103	0.3968	1.5242e + 006
	Worst	0.3331	0.4631	0.4281	0.4306	3.1513e + 006
	Std	0.0406	0.0427	0.0421	0.0612	2.4325e + 006

### Simulation contrast experiments and results analysis

Three swarm intelligence algorithms (PSO algorithm, ABC algorithm and CS algorithm) are selected to carry out the simulation contrast experiments with the proposed IGWO so as to verify its superiority on the convergence velocity and searching precision. When dimension D = 30, the simulation convergence results on the adopted testing functions are shown in Figs 12–23. when D = 100, the simulation convergence results on the adopted testing functions are shown in Figs 24–27.

**Figure 12:**
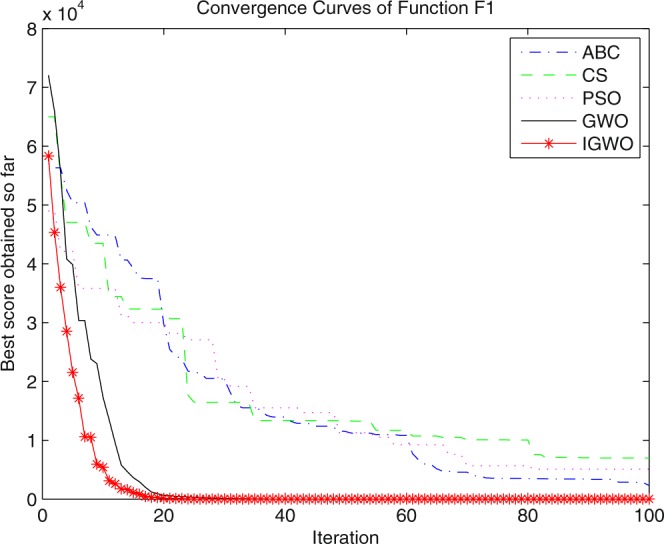
Convergence curves Function *F1*_._

**Figure 13:**
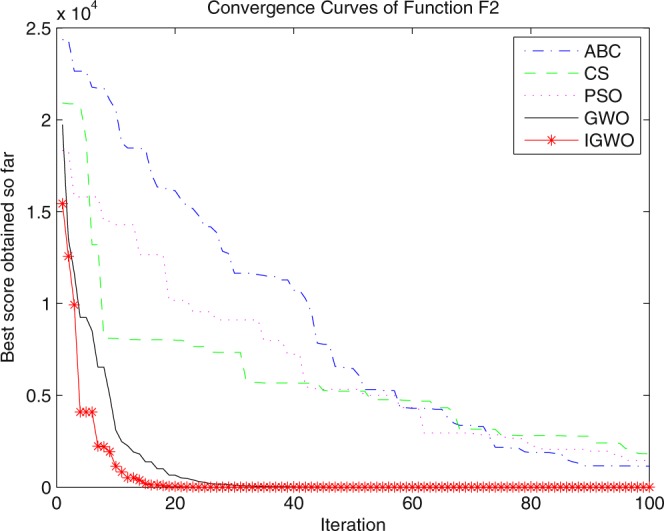
Convergence curves Function *F2*.

**Figure 14:**
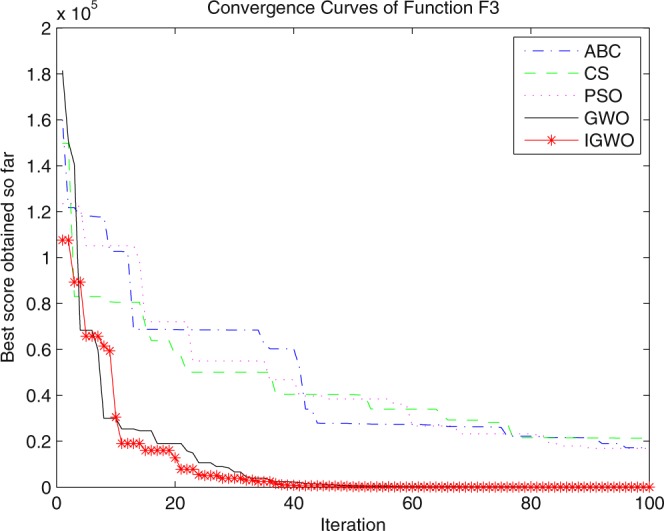
Convergence curves Function *F3*.

**Figure 15:**
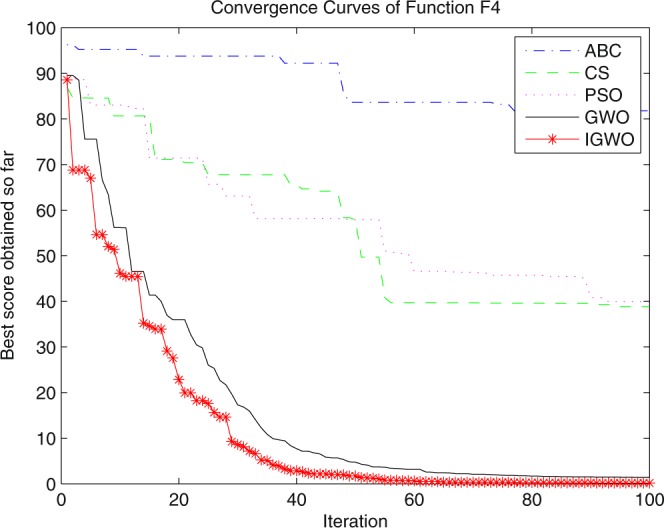
Convergence curves Function *F4*.

**Figure 16:**
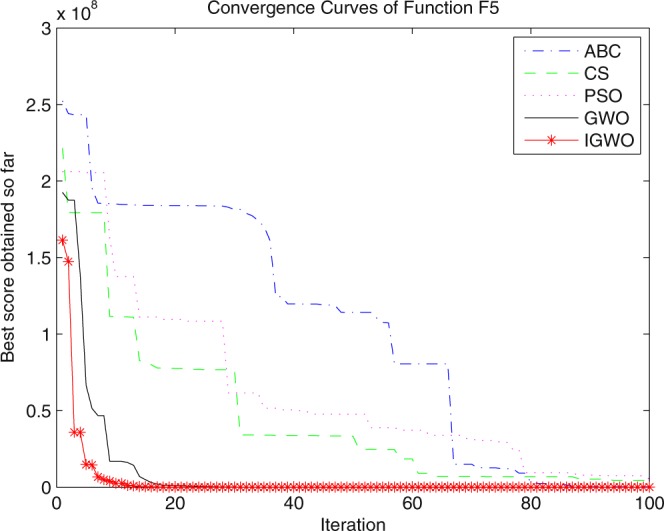
Convergence curves Function *F5*.

**Figure 17:**
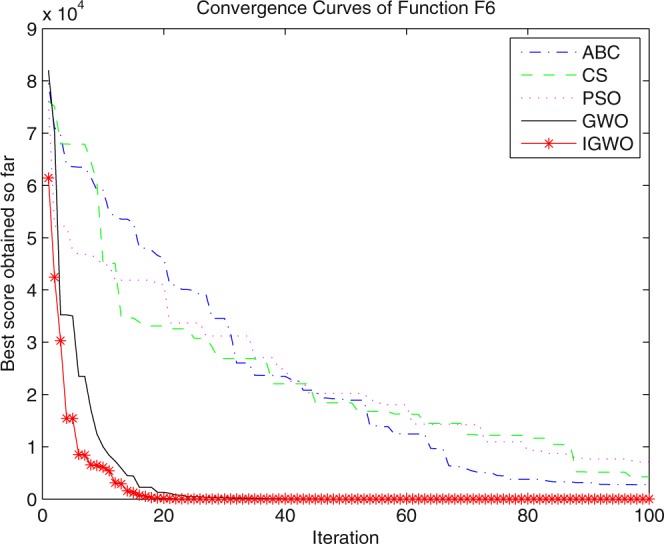
Convergence curves Function *F6*.

**Figure 18:**
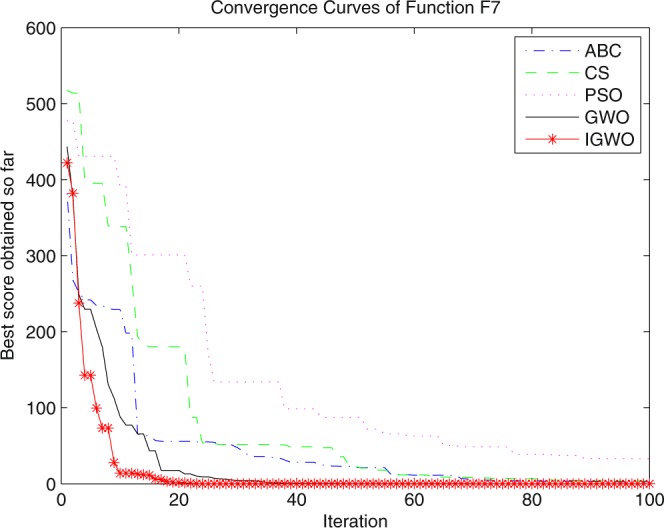
Convergence curves Function *F7*.

**Figure 19:**
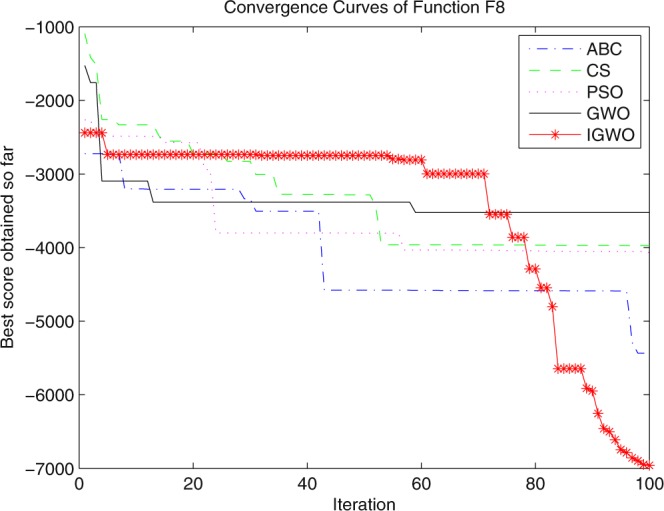
Convergence curves Function *F8*.

**Figure 20:**
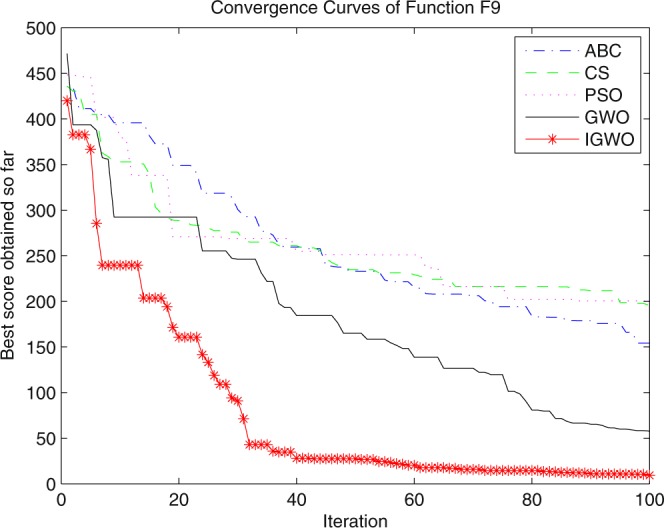
Convergence curves Function *F9*.

**Figure 21:**
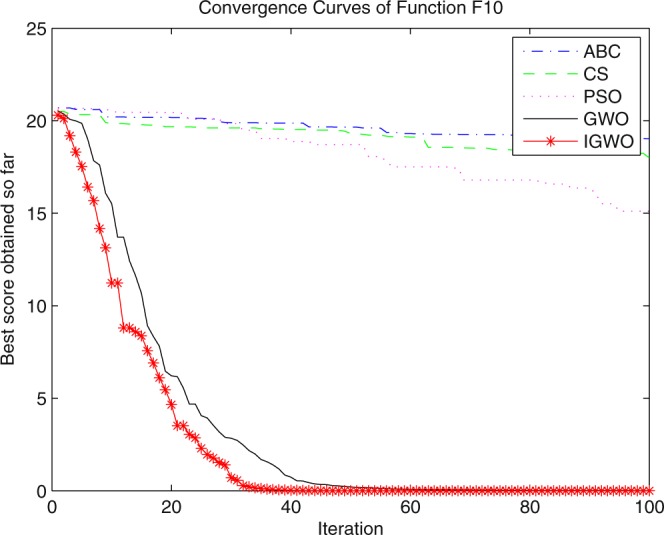
Convergence curves Function *F10*.

**Figure 22:**
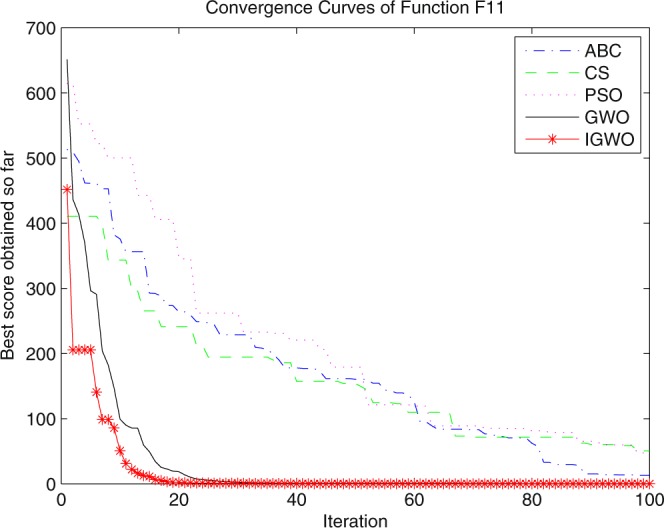
Convergence curves Function *F11*.

**Figure 23:**
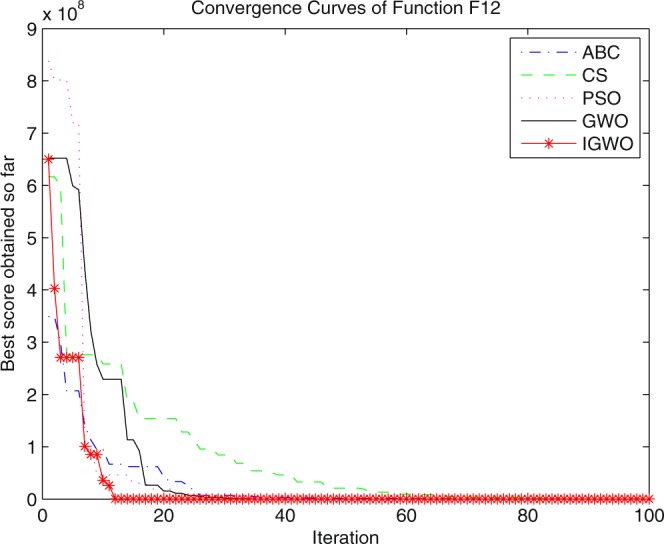
Convergence curves Function *F12*.

**Figure 24:**
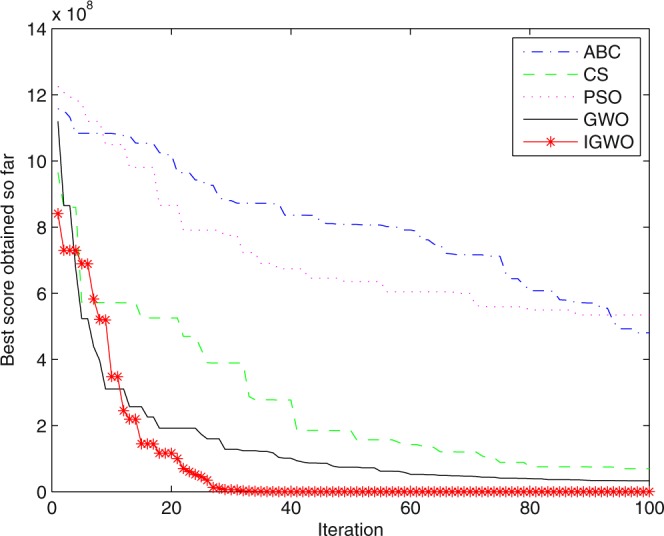
Convergence curves Function *F5* (D = 100).

**Figure 25:**
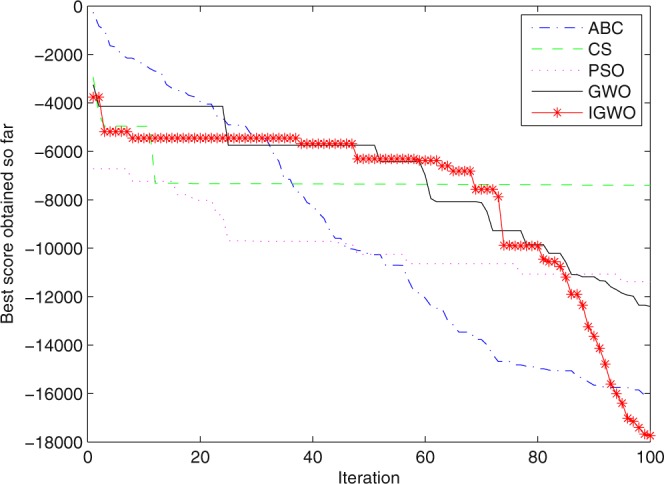
Convergence curves Function *F8* (D = 100).

**Figure 26:**
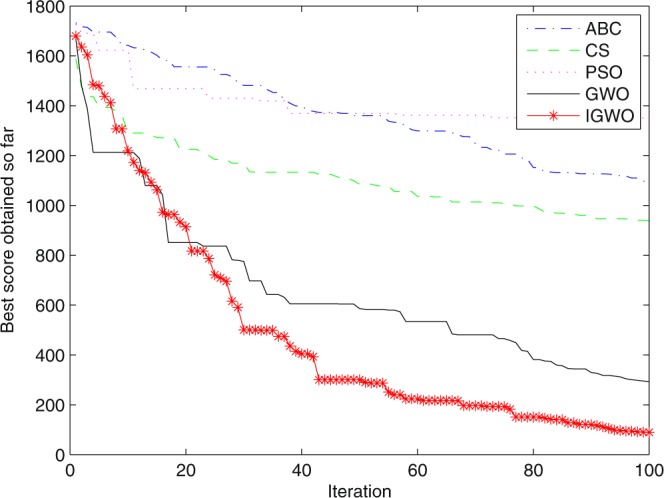
Convergence curves Function *F9* (D = 100).

**Figure 27:**
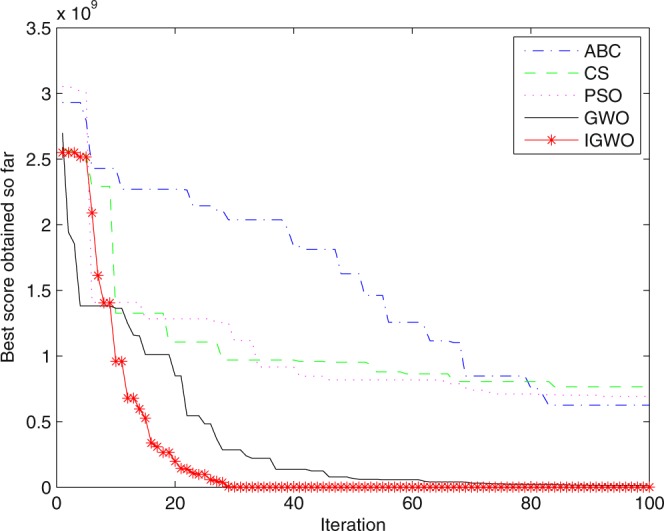
Convergence curves Function *F12* (D = 100).

It can be seen from the simulation convergence curves, IGWO has better convergence velocity and searching precision than other three algorithms (ABC, CS and PSO). Especially for function F8, F9 and F12, compared with GWO, the IGWO make their convergence speed are improved obviously. It can be seen from their surface figure that the three functions are multimodal function. So there are a lot of local minimum values within the scope of search. For IGWO, the addition of differential evolution and the SOF mechanism can improving the weakness of easily falling into the local extreme and obtain smaller function value. Thus, the convergence speeds of the three functions are improved significantly. At the same time, it suggests that the IGWO has the superiority to jump out of the local optimal.

For further validating the searching accuracy, every optimization algorithm is run independently thirty times and the best, worst and average values are recorded for the adopted twelve testing functions under the 30-dimension and 100-dimension. The maximum iterations number is *Max Max_iter* = 500. The statistical results of D = 30 and D = 100 are shown in Tables 5 and 6 respectively. Seen from the numerical results listed in Tables 5 and 6, the IGWO proposed in this paper makes optimization accuracy of 12 typical functions have a certain improvement. And the optimization accuracy of IGWO is better than that of PSO, ABC, CS algorithm under the same dimension. For the F1 function, when its dimension D = 30, its average value is raised from e-27 to e-63, relative to GWO been increased by 36 orders of magnitude; and when D = 100, its average value is raised from e-12 to e-34, relative to GWO increased by 22 orders of magnitude. For the F2 function, when D = 30, its average value has been improved 35 orders of magnitude relative to GWO; and when D = 100, relative to GWO its average value is improved 22 orders of magnitude. For function F9 and F11, when D = 30 and D = 100, IGWO respectively search to their ideal minimum value 0. The optimization performance for other testing functions all has been improved.

**Table 5:** Numerical statistics results of D = 30.

Function		IGWO	GWO	PSO	ABC	CS
**F1**	Best	**3.9273e** **−** **069**	2.0291e **−** 029	5.1422	1.9075e **−** 005	5.3943
	Ave	1.1783e **−** 064	1.0402e **−** 027	157.3150	7.3230e **−** 004	17.2298
	Worst	1.2505e **−** 062	6.8810e **−** 027	1.3957e + 003	0.0061	39.5997
	Std	5.5606e-063	1.4561e **−** 027	618.8854	5.5855e **−** 004	10.6054
**F2**	Best	**7.6878e** **−** **068**	2.0613e **−** 030	2.8795	6.2539e **−** 006	0.0791
	Ave	3.2484e **−** 064	6.5600e **−** 029	108.5533	2.1544e **−** 004	0.2208
	Worst	8.4385e **−** 063	4.8634e **−** 028	1.0320e + 003	0.0019	0.9046
	Std	4.1566e **−** 066	1.5256e **−** 028	113.0001	1.0957e **−** 004	0.1073
**F3**	Best	**1.7846e** **−** **015**	2.6272e **−** 008	2.6128e + 003	19.2563	82.0828
	Ave	3.6220e **−** 0010	1.4089e **−** 005	5.8899e + 003	214.0276	388.8103
	Worst	5.5878e **−** 008	1.7721e **−** 004	1.5150e + 004	512.2673	892.5678
	Std	4.3091e **−** 008	5.1567e **−** 005	2.7730e + 003	353.0218	753.0257
**F4**	Best	**4.0775e** **−** **014**	8.7151e **−** 008	10.5604	34.6791	8.2559
	Ave	4.2932e **−** 013	1.0129e **−** 006	19.4012	65.4533	11.5000
	Worst	7.1302e **−** 011	7.7390e **−** 006	30.8453	82.1336	16.9572
	Std	3.7329e **−** 012	3.5107e **−** 007	4.9307	7.6916	1.2514
**F5**	Best	**23.1787**	25.7961	582.0313	40.4974	111.9810
	Ave	25.3873	28.0325	1.7004e + 004	88.0783	600.0555
	Worst	28.5692	28.7800	8.3090e + 004	502.2101	2.2452e + 003
	Std	0.6997	0.9258	4.9416e + 004	29.4371	274.0168
**F6**	Best	**7.5296e** **−** **005**	0.0063	7.6943	3.4009	8.6597
	Ave	0.6583	0.8993	300.3189	8.3567	24.2355
	Worst	2.1169	1.5192	1.1916e + 003	23.0030	75.3820
	Std	0.2917	0.4055	374.9472	3.2274e **−** 004	12.7746
**F7**	Best	**1.2695e** **−** **004**	4.5944e **−** 004	0.1186	0.0319	0.0177
	Ave	7.8361e **−** 004	0.0022	0.6129	0.0528	0.0621
	Worst	0.0017	0.0064	2.2257	0.8170	0.1222
	Std	3.7904e **−** 004	8.4619e **−** 004	0.3202	0.0026	0.0159
**F8**	Best	**−3.9429e + 004**	−6.1310e + 003	−4.1322e + 003	−1.1681e + 003	−6.6092e + 003
	Ave	−9.0105e + 003	−3.6813e + 003	−3.4422e + 003	−1.1294e + 003	−5.9192e + 003
	Worst	−3.3482e + 003	−2.9262e + 003	−2.9516e + 003	−1.0866e + 003	−5.2163e + 003
	Std	−1.7912e + 003	−958.0854	3.2960e + 003	205.0614	351.8938
**F9**	Best	**0**	1.1369e **−** 013	46.3981	5.1504	53.0900
	Ave	1.0783	3.2143	92.6839	9.0702	73.5890
	Worst	12.1696	15.8356	152.7472	13.8016	103.0006
	Std	1.5038	4.8809	22.7852	2.5126	14.3049
**F10**	Best	**1.5099e** **−** **017**	7.5495e **−** 013	2.0863	1.6725	4.1810
	Ave	1.2204e **−** 016	1.0048e **−** 012	5.7135	5.0268	7.0560
	Worst	2.2204e **−** 014	1.4655e **−** 013	10.5829	11.2607	14.1411
	Std	4.3110e **−** 015	1.4373e **−** 014	2.4526	1.7819	2.6353
**F11**	Best	**0**	0	1.1890	3.3792e **−** 005	1.0869
	Ave	0.0016	0.0048	7.4790	0.0649	1.2128
	Worst	0.0147	0.0286	26.8061	0.1863	1.5050
	Std	0.0074	0.0114	3.9852	0.0431	0.1070
**F12**	Best	**0.0065**	0.0188	1.4887	1.8107	1.0945
	Ave	0.0481	0.0594	73.9029	4.5941	3.6666
	Worst	0.1091	0.0819	2.0290e + 003	6.8171	6.5449
	Std	0.0151	0.0419	4.4614	0.3128	0.4337

**Table 6:** Numerical statistics results of D = 100.

Function		IGWO	GWO	PSO	ABC	CS
**F1**	Best	**1.5561e** **−** **035**	3.4355e **−** 013	3.7127e + 004	122.6512	3.4168e + 003
	Ave	9.5901e **−** 034	1.4097e **−** 012	4.9247e + 004	4.0245e + 003	5.7296e + 003
	Worst	1.8708e **−** 032	3.6831e **−** 012	6.5987e + 004	1.0067e + 004	8.6850e + 003
	Std	7.1710e **−** 034	2.8268e **−** 012	8.9305e + 003	1.8523e + 003	912.3910
**F2**	Best	**2.4553e** **−** **036**	1.6794e **−** 013	2.6127e + 004	91.5939	1.3798e + 003
	Ave	7.1368e **−** 034	1.0558e **−** 012	3.7423e + 004	3.9368e + 003	2.0391e + 003
	Worst	5.9594e **−** 033	4.7932e **−** 012	5.6874e + 004	1.1487e + 004	3.0315e + 003
	Std	1.6942e **−** 034	3.1877e **−** 012	7.9858e + 003	2.1492e + 003	514.5256
**F3**	Best	**23.6476**	65.1383	1.1274e + 005	2.9201e + 004	3.0971e + 005
	Ave	679.3675	818.9336	1.8885e + 005	1.2577e + 005	5.4593e + 005
	Worst	2.5006e + 003	5.5568e + 003	3.9436e + 005	8.1136 + e005	7.4755e + 005
	Std	1.0607e **−** 009	3.0654e **−** 005	2.6625e + 03	1.6236e + 004	1.7767e + 004
**F4**	Best	**0.0018**	0.0607	43.1594	90.3059	22.7549
	Ave	0.0382	0.9562	54.6512	95.0132	29.3341
	Worst	0.6247	3.1841	66.4211	98.3757	39.2722
	Std	0.4072	0.6513	4.8263	1.6038	2.8819
**F5**	Best	**95.8954**	96.8501	2.6394e + 007	8.4200e + 003	4.2931e + 005
	Ave	96.8157	98.0217	5.7355e + 007	7.8186e + 005	1.0015e + 006
	Worst	98.5244	98.5207	1.3108e + 008	9.9299e + 006	2.4179e + 006
	Std	0.6162	0.6708	1.3951e + 007	1.9970e + 005	3.2088e + 005
**F6**	Best	**7.4830**	9.7480	2.1793e + 004	210.6135	4.2828e + 003
	Ave	9.3215	12.1379	3.4705e + 004	4.4723e + 003	5.7352e + 003
	Worst	12.6824	12.7860	4.6567e + 004	1.1812e + 004	8.5045e + 003
	Std	0.7722	0.9744	4.9192e + 003	2.5176e + 003	1.0449e + 003
**F7**	Best	**3.5264e** **−** **004**	0.0026	28.9078	7.9176	0.4508
	Ave	0.0024	0.0066	92.8878	10.3561	0.7003
	Worst	0.0043	0.0130	236.6874	11.2352	1.0116
	Std	7.0880e **−** 004	0.0021	47.0314	4.3578	0.1182
**F8**	Best	**−8.2275e + 004**	−1.9447e + 004	−1.6506e + 004	−1.1603e + 004	−1.2130e + 004
	Ave	−6.0161e + 004	−1.5641e + 004	−5.5154e + 003	−1.0941e + 004	−1.0789e + 004
	Worst	−5.0981e + 003	−5.5875e + 003	−1.7081e + 003	−2.7028e + 004	−9.3055e + 003
	Std	672.4919	4.6259e + 003	4.4865e + 003	582.5893	643.1070
**F9**	Best	**0**	4.8431e **−** 011	722.3155	243.0193	350.2439
	Ave	1.6643	9.4635	877.8493	293.0193	428.6253
	Worst	12.7963	30.0252	1.0625e + 003	342.8732	493.4137
	Std	2.6605	6.4071	86.9610	21.9504	38.0722
**F10**	Best	**6.4837e** **−** **014**	5.9873e **−** 008	15.8294	8.2314	10.9187
	Ave	7.7153e **−** 014	1.1140e **−** 007	17.0826	11.2388	13.3370
	Worst	9.3259e **−** 014	2.2161e **−** 007	20.7326	15.7326	17.6444
	Std	6.0990e **−** 015	6.2626e **−** 008	0.6998	1.6529	1.9379
**F11**	Best	**0**	1.4655e **−** 013	301.0689	2.2155	38.0069
	Ave	0.0028	0.0065	430.3317	29.6147	54.1621
	Worst	0.0254	0.0270	560.6552	76.0269	75.9006
	Std	0.0076	0.0110	52.2074	15.3837	11.3528
**F12**	Best	**0.1056**	0.2154	8.6436e + 006	20.1113	20.6985
	Ave	0.1551	0.3968	3.2730e + 007	1.3921e + 004	6.5202e + 003
	Worst	0.3331	0.4306	9.5779e + 007	1.4818e + 005	8.1864e + 004
	Std	0.0406	0.0612	4.3908e + 007	3.2646e + 004	0.8511

Thus, the simulation experiments results, including convergence curves and statistics data, show that the proposed improved grey wolves optimizer has a better convergence rate and optimization performance. According to the improvement above, three reasons can be summarized: Firstly, the adding of evolution operation is able to increase the diversity of wolves, that is to say to increase the solution diversity so as to make the algorithm jump out the local extreme. Secondly, the selection method of parents of DE and dynamic scaling factor *F* can make the algorithm has a good exploration ability in the early search stage and has a good exploitation ability in the later search stage, therefore, both search precision and convergence speed are improved. In addition, the adding of SOF wolf updating mechanism can also decrease the probability of the algorithm falling into the local extreme.

## Conclusions

For achieving the proper compromise between exploration and exploitation, further accelerate the convergence and increase the optimization accuracy of GWO, an improved grey wolf optimizer (IGWO) is proposed in this paper. The biological evolution and SOF principle in nature are added to the standard GWO. The simulation experiments are carried out by adopting twelve typical function optimization problems. The simulation results show the proposed IGWO has better convergence velocity and optimization performance than DE algorithm, PSO algorithm, ABC algorithm and CS algorithm. On the one hand, the adoption of evolution operation can increase the wolves diversity and make the algorithm has a good exploration ability in the early searching stage and has a good exploitation ability in the later search stage. On the other hand, the adoption of SOF wolf updating mechanism can decrease the probability of falling into the local optimum. In the future work, we will carry out similar hybridisation of other swarm intelligent optimization algorithms, such as dragonfly algorithm (DA), ant lion optimizer (ALO), multi-verse optimizer (MVO), coral reefs optimization (CRO) algorithm, etc.

## Data Availability

There are no data available for this paper.
